# A Novel High-Energy Vacuum Ultraviolet Light Photofunctionalization Approach for Decomposing Organic Molecules around Titanium

**DOI:** 10.3390/ijms24031978

**Published:** 2023-01-19

**Authors:** Toshikatsu Suzumura, Takanori Matsuura, Keiji Komatsu, Takahiro Ogawa

**Affiliations:** Division of Regenerative and Reconstructive Sciences and Weintraub, Center for Reconstructive Biotechnology, UCLA School of Dentistry, Los Angeles, CA 90095-1668, USA

**Keywords:** photofunctionalization, osseointegration, dental and orthopedic implants, hydrocarbon, biological aging of titanium

## Abstract

Titanium undergoes biological aging, represented by increased hydrophobicity and surface accumulation of organic molecules over time, which compromises the osseointegration of dental and orthopedic implants. Here, we evaluated the efficacy of a novel UV light source, 172 nm wavelength vacuum UV (VUV), in decomposing organic molecules around titanium. Methylene blue solution used as a model organic molecule placed in a quartz ampoule with and without titanium specimens was treated with four different UV light sources: (i) ultraviolet C (UVC), (ii) high-energy UVC (HUVC), (iii) proprietary UV (PUV), and (iv) VUV. After one minute of treatment, VUV decomposed over 90% of methylene blue, while there was 3-, 3-, and 8-fold more methylene blue after the HUVC, PUV, and UVC treatments, respectively. In dose-dependency experiments, maximal methylene blue decomposition occurred after one minute of VUV treatment and after 20–30 min of UVC treatment. Rapid and effective VUV-mediated organic decomposition was not influenced by the surface topography of titanium or its alloy and even occurred in the absence of titanium, indicating only a minimal photocatalytic contribution of titanium dioxide to organic decomposition. VUV-mediated but not other light source-mediated methylene blue decomposition was proportional to its concentration. Plastic tubes significantly reduced methylene blue decomposition for all light sources. These results suggest that VUV, in synergy with quartz ampoules, mediates rapid and effective organic decomposition compared with other UV sources. This proof-of-concept study paves the way for rapid and effective VUV-powered photofunctionalization of titanium to overcome biological aging.

## 1. Introduction

Dental implants, orthopedic implants, and many other prosthetic components are made of titanium-based materials, especially commercially pure titanium or titanium alloys. Titanium and titanium alloys show excellent biocompatibility and osseointegration compared with other materials [1,2,3,4,5,6,7,8,9,10,11,12,13,14,15,16,17,18,19]. However, it has recently been shown that titanium undergoes biological aging, characterized by the loss of hydrophilicity of titanium surfaces over time [20,21,22,23,24,25,26,27], which correlates with their decreasing ability to osseointegrate. Aging is also associated with the accumulation of hydrocarbons on titanium surfaces, which further contributes to a loss of osseointegration [20,21,22,23,28,29,30]. Biological aging is an inevitable and undesirable process for all types of titanium and titanium alloys [28,29,31,32,33,34,35,36]. One effective way to increase the osseointegration of titanium is to remove carbon molecules from its surface before placing it into bone [37,38,39,40,41,42,43,44,45,46,47].

UV photofunctionalization or UV activation was developed to overcome the biological aging of titanium [20,23,34,35,45,46,47,48,49,50,51,52,53,54,55,56,57,58,59,60,61,62,63,64,65,66,67,68,69,70,71,72,73,74,75]. The treatment of titanium with UV light decomposes and removes carbon-containing molecules, reducing the atomic percentage of carbon from 40–75% to 15–20% [20,48,49,50,76]. Treated titanium surfaces consequently regain hydrophilicity. UV-treated, decarbonized titanium surfaces then recruit osteogenic cells, facilitate cell attachment and adhesion, promote osteoblastic proliferation and differentiation, and accelerate and increase bone formation around titanium [21,23,24,48,49,57,62,77,78]. However, there remain three major challenges to the practical and effective use of UV photofunctionalization in clinical practice [50,51,53,79,80]. First, UV treatment has not been optimized; there is still uncertainty about the optimal UV dose or if there even is an optimal dose. Second, the treatment time remains long; despite significant advances in photo-technology, the proposed UV treatment time is still 48 h [48,49,81], 20 min [38,44,51,52,53,63,80,82,83,84], or 12 min [33,37,42] depending on the light source; these intervals which may be acceptable for research but are all too long for routine clinical use. Third, current UV treatment can only be achieved when titanium specimens have been removed from their sterile containers due to only very limited penetration of UV light into these containers [53,60,63,85,86]. Implantable medical devices, including dental and orthopedic implants, pins, plates, and screws, are packed in sterilized plastic or metal containers and are expected to be immediately delivered to patients directly from these containers. Exposing the devices to the atmosphere or surroundings during UV treatment carries a risk of contamination. Unfortunately, the higher the UV photon energy is, the lower the penetration through current container materials is.

UV photofunctionalization was originally described using a bactericidal UV lamp for 48 h [49]. The lamp emitted UV-C (200–280 nm wavelength), with a peak around 254 nm. The UV-mediated decomposition of carbon-containing molecules is thought to occur via two mechanisms: direct decomposition or through photocatalytic decomposition via titanium dioxide [49,50,87,88,89,90]. In the direct UV decomposition mechanism, UV light breaks atomic bonds according to the strength of photon energy; for instance, a relatively weak, single oxygen–carbon (C-O) bond can be broken by low-energy UV-A (374 nm wavelength), while double oxygen bonds (O=O) can be dissociated by high-energy UV-B (243 nm wavelength). Another mechanism of direct UV-mediated organic decomposition is the generation of oxygen radicals. For example, UV-C produces radicals through ozone, which attacks organic molecules, with dissociated molecules such as CO_2_, H_2_O, and O_2_ being released into the atmosphere. In the second mechanism, photocatalytic organic decomposition is induced by two reactive oxygen species produced on titanium dioxide [89,90,91,92]. UV energy releases free electrons (e^−^) and forms a positive hole (h^+^) on titanium dioxide as a result. Free electrons (e^−^) react with atmospheric oxygen (O_2_) to produce superoxide anions (O_2_^.−^), which in turn dissolve organic molecules through oxidation. Positive holes (h^+^) react with atmospheric water (H_2_O) to generate hydroxy radicals (^.^OH), which act in a manner similar to that of superoxide anions.

Recent technical advances have resulted in a high-energy, low-pressure mercury vapor lamp that reduced UV treatment to 20 min to generate a similar effect to 48 h bactericidal UV treatment, and the latest UV device uses a proprietary wavelength for 12 min treatment. Here, we tested the potential of a novel, xenon excimer lamp emitting 172 nm wavelength vacuum UV (VUV; defined as UV < 200 nm) to decompose organic molecules around titanium. Specifically, we determined whether the combined use of 172 nm VUV and a quartz ampoule [93,94] as a specimen container overcame the above-mentioned three challenges. Methylene blue (C_16_H_18_ClN_3_S), a benzene ring, hydrocarbon-containing molecule, was used as a model organic molecule. We tested the device on microroughened commercially pure titanium surfaces (most commonly used in dental and orthopedic implants), machined titanium surfaces, and machined titanium alloy surfaces. The efficacy of organic decomposition was assessed according to (i) direct decomposition induced by UV light and (ii) photocatalytic decomposition induced by UV-excited titanium dioxide. Finally, we attempted to optimize the UV dose by identifying a plateau in efficacy.

## 2. Results

### 2.1. Surface Characteristics of Titanium Materials

**Figure 1:**
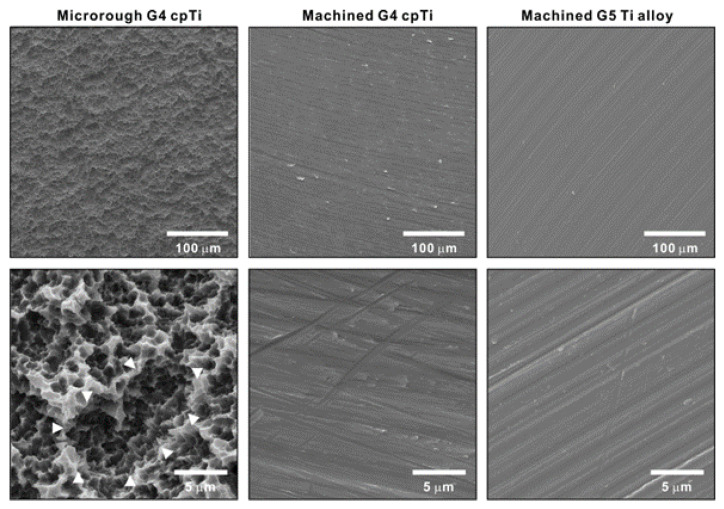
Surface morphology of titanium specimens used in this study. Low (**upper** panels)- and high (**lower** panels)-magnification SEM images of three different specimens.

The surface morphology and hydrophilic/hydrophobic state of three titanium test materials were examined. The surface of acid-etched commercially pure grade 4 titanium was significantly rougher than that of machined grade 4 titanium and machined grade 5 titanium alloy according to low-magnification SEM (Figure 1). The high-magnification images showed that the acid-etched commercially pure titanium was evenly roughened, with a combination of scattered supra-micron concavities (arrowheads in lower-left panel) and micro-pits over the surface. The machined surfaces, regardless of titanium type, had no defined topography except for linear traces from machine milling.

**Figure 2:**
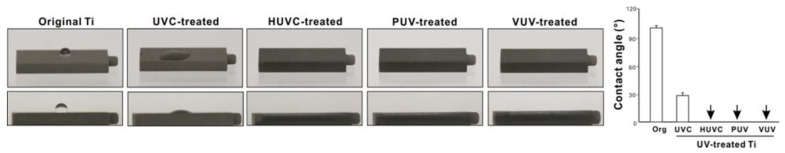
Hydrophilic/hydrophobic state of acid-etched microroughened titanium surfaces before and after UV treatment exposed to four different UV light sources for one minute: UVC from a low-pressure mercury lamp (UVC); high-energy UVC (HUVC); a UV device with a proprietary protocol (PUV); and 172 nm vacuum UV (VUV). Birds-eye and side-view photographs of a 3 µL drop of ddH_2_O placed on microroughened titanium specimens, and a histogram showing contact angle measurements. Arrows indicate 0°.

The hydrophilicity of acid-etched microroughened commercially pure titanium was examined before and after UV treatment for 1 min using four different light sources: (i) ultraviolet C (UVC), (ii) high-energy UVC (HUVC), (iii) proprietary UV (PUV), and (iv) VUV (Figure 2). Original titanium surfaces were hydrophobic, with a contact angle > 90°. All UV treatments converted the surfaces to hydrophilic (defined as a contact angle < 30°), the degree varied according to the UV source, with the HUVC, PUV, and VUV treatments all resulting in a contact angle of 0° and UVC in a contact angle of ~30%.

### 2.2. Organic Molecule Decomposition Induced by Different UV Light Sources

**Figure 3:**
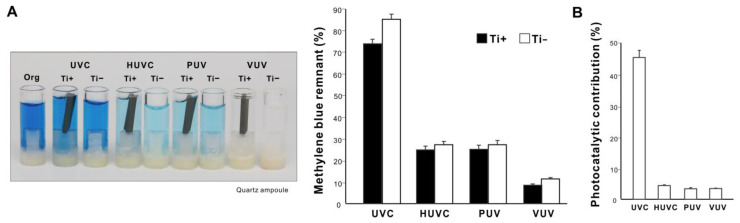
UV-mediated decomposition of methylene blue induced by various UV sources. (**A**) Photographs of the original methylene blue solution in a quartz ampoule together with solutions after UV treatment presented side-by-side for comparison. Four different UV light sources were used for one minute: (1) UVC from a low-pressure mercury lamp (UVC); (2) high-energy UVC (HUVC); (3) a UV device with a proprietary protocol (PUV); and (4) 172 nm vacuum UV (VUV). Methylene blue was UV-treated with or without a microroughened titanium specimen. A histogram showing remnant methylene blue after UV treatment (%). (**B**) Photocatalytic contribution (%) calculated as the difference in the amount of decomposed methylene blue with and without a titanium specimen.

We next compared the ability of different UV sources to decompose organic molecules. Microroughened commercially pure titanium was used as a representative test material and methylene blue as a model organic molecule. Methylene blue placed in a quartz ampoule was treated with UV light for 1 min with or without a titanium specimen. All UV treatments significantly decomposed methylene blue regardless of the light source; however, the amount of remaining methylene blue varied considerably (*p* < 0.001, two-way ANOVA), with UVC being the highest and VUV the lowest (Figure 3A) (*p* < 0.001, Bonferroni-corrected). With a titanium specimen, one minute of VUV treatment decomposed over 90% of methylene blue, versus ~25% for UVC and ~75% for HUVC and PUV. UV treatment with a titanium specimen decomposed more methylene blue than without a titanium specimen for all light sources (*p* < 0.05, Bonferroni-corrected). The difference in methylene blue decomposition with and without a titanium specimen was represented as photocatalytic contribution (%), which was significantly lower for HUVC, PUV, and VUV (<5%) than for UVC (~45%) (*p* < 0.001; Figure 3B).

### 2.3. Dose Dependency of Organic Decomposition Induced by VUV and UVC

**Figure 4:**
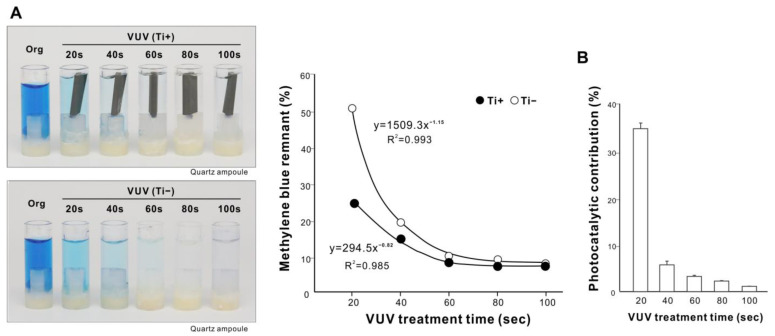
Dose dependency and optimization of VUV-mediated organic decomposition. (**A**) Photographs of the original methylene blue solution in a quartz ampoule together with solutions after VUV treatment for various treatment times from 20 to 100 s. The remaining methylene blue was quantified and plotted against UV treatment time; it showed a highly fitted, negative exponential curve. (**B**) Photocatalytic contribution (%) calculated as the difference in the amount of decomposed methylene blue with and without a microroughened titanium specimen.

After establishing that VUV had the greatest methylene blue decomposition efficacy, we tested for dose dependency by varying the treatment time. With a titanium specimen in a quartz ampoule, the amount of remnant methylene blue significantly decreased with the increase in treatment time up to 60 s to then plateau at about 10%, fitting a negative exponential curve with a remarkably high coefficient of determination (Figure 4A). VUV treatment without a titanium specimen showed a similar dose dependency, but with a lower efficacy of decomposition. The photocatalytic contribution decreased with longer treatment time (Figure 4B). Thus, the maximum efficacy of organic decomposition induced by VUV was achieved with an optimized treatment time of 1 min.

**Figure 5:**
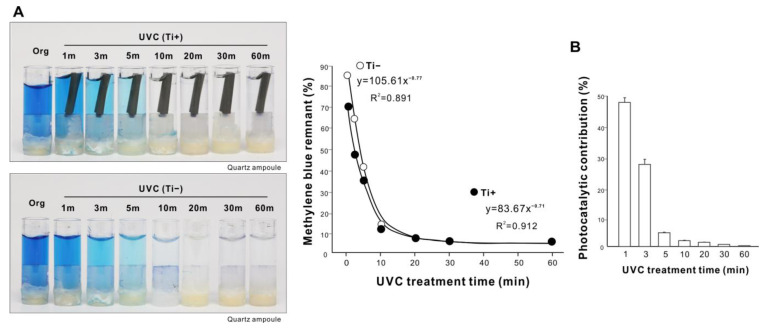
Dose dependency of UVC organic decomposition. (**A**) Photographs of the original methylene blue solution in a quartz ampoule together with solutions after UVC treatment for various treatment times from 1 to 60 min. The remaining methylene blue was quantified and plotted against UV treatment time; it showed a highly fitted, negative exponential curve. (**B**) Photocatalytic contribution (%) calculated as the difference in the amount of decomposed methylene blue with and without a microroughened titanium specimen.

We further tested for the presence of UV dose dependency using UVC. Again, the length of UVC treatment was proportional to the degree of methylene blue decomposition (Figure 5A), plateauing at 20–30 min along a negative exponential curve. With both UV sources, UV treatment was unable to completely decompose methylene blue within the treatment time tested, suggesting a general limit to UV-mediated organic decomposition. Photocatalysis induced by a titanium specimen contributed less as treatment time increased (Figure 5B).

### 2.4. Effect of Quartz Ampoules on UV-Mediated Organic Decomposition

**Figure 6:**
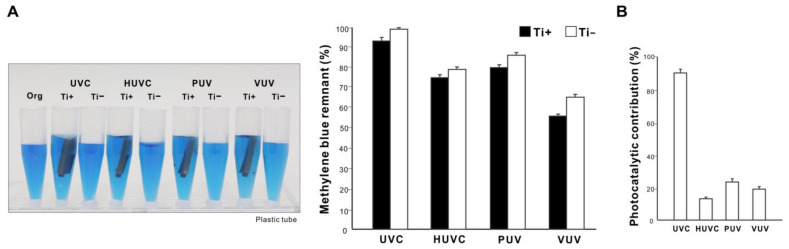
UV-mediated decomposition of methylene blue in plastic tubes. (**A**) Photographs of the original methylene blue solution in a laboratory-grade plastic tube together with solutions after UV treatment presented side-by-side for comparison. Four different UV light sources were used for one minute, as in Figure 3. UV treatment was conducted with or without a microroughened titanium specimen. A histogram showing remnant methylene blue after UV treatment (%). (**B**) Photocatalytic contribution (%) calculated as the difference in the amount of decomposed methylene blue with and without a titanium specimen.

We next compared the impact of quartz ampoules and laboratory-grade plastic tubes on organic decomposition efficacy by assessing methylene blue decomposition using one minute of UV treatment with or without a titanium specimen. Methylene blue decomposition was greater in quartz ampoules than plastic tubes for all UV treatments (Figure 3A and Figure 6A); for instance, there was >90% residual methylene blue in plastic tubes after UVC treatment compared with 75% in quartz ampoules. VUV showed the highest efficacy of decomposition among the UV sources, with approximately 55% remnant methylene blue, although significantly more than the 9% seen in the quartz ampoule (Figure 3A and Figure 6A). The photocatalytic contribution was significantly higher in plastic tubes than in quartz ampoules (Figure 3B and Figure 6B), with the majority of methylene blue decomposition in plastic tubes being due to titanium photocatalysis during UVC decomposition.

### 2.5. Effects of Different Titanium Materials and Surfaces

**Figure 7:**
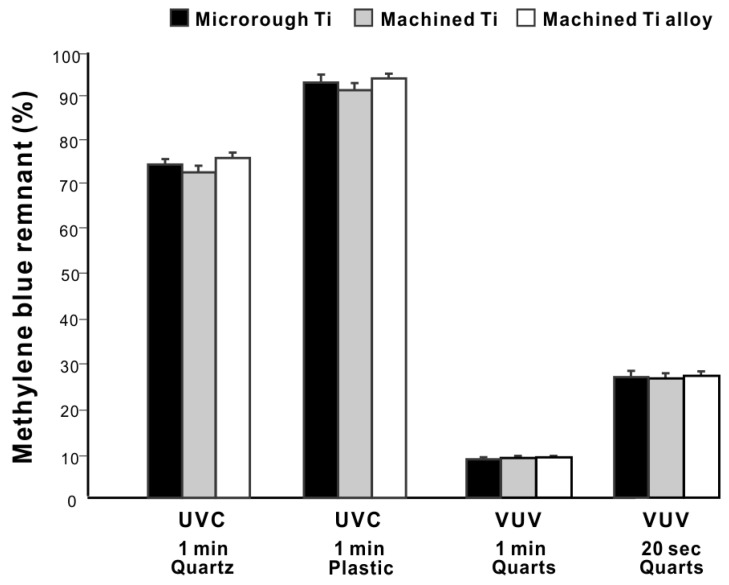
Effects of different titanium materials and surfaces on UV-mediated organic decomposition. UV treatment was conducted under the selected conditions with either microroughened commercially pure titanium, machined commercially pure titanium, or machined grade 5 titanium alloy. A histogram showing remnant methylene blue after UV treatment (%).

We next determined if the organic decomposition efficacy differed between titanium specimens using UV treatment under four representative conditions (1 min UVC in quartz ampoules, 1 min UVC in plastic tubes, 1 min VUV in quartz ampoules, and 1 min VUV in plastic tubes). There was no significant difference in efficacy between microroughened commercially pure titanium, machine-surfaced commercially pure titanium, and machine-surfaced titanium alloy (Figure 7), indicating that the photocatalytic activity was not significantly affected by different titanium materials or surfaces.

### 2.6. Load Testing of UV-Mediated Organic Decomposition

**Figure 8:**
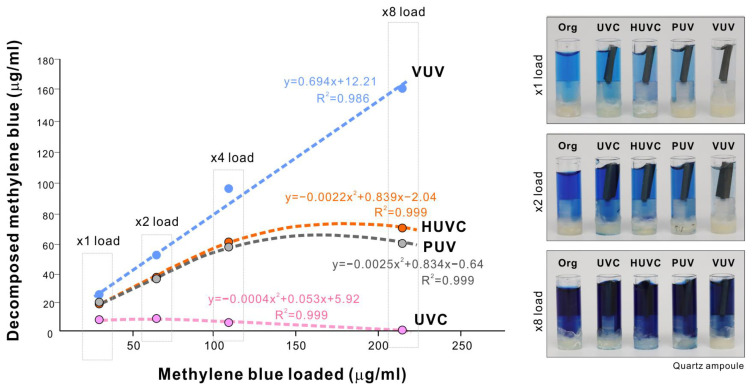
Load testing of organic decomposition induced by four different UV light sources. Methylene blue at four different concentrations (1× to 8×) in a quartz ampoule was treated with four different UV light sources for 1 min. Remnant methylene blue is plotted against the methylene blue concentration along with results of the regression analysis. Note that a linear positive correlation was only found for VUV, while the other UV sources fitted a polynomial equation. Representative photographs of the methylene blue solutions are presented.

We next load-tested the four different UV sources to determine their tolerance to or capacity of organic decomposition. Using a one-minute treatment protocol, various concentrations of methylene blue were decomposed in a quartz ampoule. The UVC treatment did not increase the amount of decomposed methylene blue, rather declining it to near zero as the methylene blue concentration increased (Figure 8). The HUVC and PUV treatments showed a load-dependent increase up to x4 load, followed by a plateau or decline. The regression analysis fitted a polynomial equation for UVC, HUVC, and PUV treatments, with very high coefficients of correlation. In contrast, methylene blue decomposition linearly increased with the increase in VUV treatment load, with greater concentrations of methylene blue resulting in greater removal. These results indicate unlimited tolerance of VUV-mediated organic decomposition compared with a maximal capacity for other UV sources within the range of loading tested in this study.

## 3. Discussion

In pursuit of advancing UV photofunctionalization for routine application to dental and orthopedic implants, here, we conducted a series of experiments to optimize the speed and efficacy of organic molecule decomposition induced by UV light sources, especially a novel, xenon excimer lamp emitting 172 nm wavelength VUV. VUV showed particularly high efficacy, which here we tested for the first time on titanium implants [95,96,97,98,99]. Only 9% of methylene blue remained after one minute of VUV treatment, while 25% remained after HUVC and PUV treatments, a 2.8-fold difference in efficacy. UVC was the least effective light source tested, with 74% of methylene blue remaining after treatment. HUVC and PUV have been commercialized for photofunctionalizing dental implants using 20 and 12 min treatment protocols, respectively [33,52,100,101]. The substantial improvement offered by VUV demonstrated here strongly suggests that this approach requires further clinical validation. Furthermore, our dose-dependency experiments revealed that VUV achieved maximum organic decomposition in one minute, considerably faster than existing protocols. This optimization (1) provides empirical evidence on which to further refine the UV photofunctionalization protocol for bone integration and (2) drastically expedites the processing time for clinical application.

Robust VUV-mediated organic decomposition was only possible with the synergy afforded by the use of quartz ampoules. VUV decays proportionally with the increase in the distance from the source due to progressive absorption by the atmosphere or any intervening materials [102,103,104,105,106]. Unfortunately, higher-energy UV light with shorter wavelength decays even more when passing through materials. Furthermore, UV light decays by 60 to 100% through plastic, depending on its thickness and molecular structure, and even glass nearly completely absorbs UV light <300 nm due to impurities such as iron oxide. Indeed, we found that methylene blue decomposition was significantly compromised by plastic, but despite this, VUV retained high performance. As mentioned in the Introduction section, implantable medical devices, including dental and orthopedic implants, are packed in sterilized plastic or metal containers. The present result, revealing excellent UV permeability of quartz ampoules, provides a novel strategy and solution for packaging medical implants, compatible with photofunctionalization. Synthetic quartz can be shaped freely as shown in the present study and is widely used in the industrial field. Now that the clinically viable one-minute protocol has been introduced for effective and rapid photofunctionalization, re-designing implant packages is justified. Thus, exploiting a synergy of VUV and quartz ampoules could open the door to a new standard of implant therapy.

**Figure 9:**
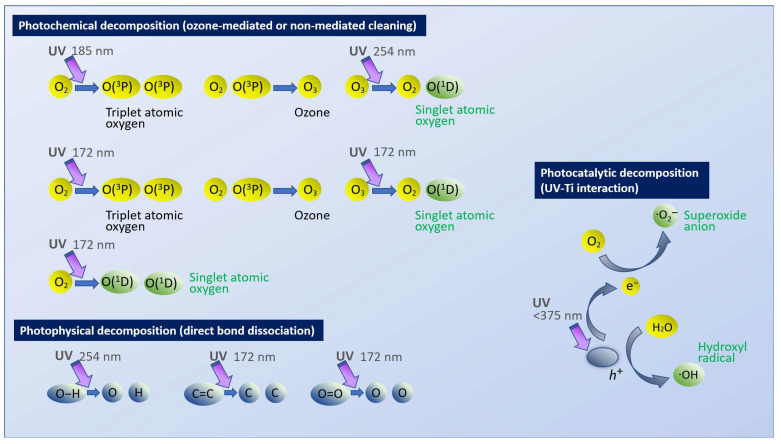
Schematic of three different mechanisms of UV-mediated organic decomposition. During photochemical and photocatalytic decomposition, the generated reactive oxygens (highlighted in green) attack organic molecules. Note that 172 nm VUV light has both photochemical and photophysical advantages, enabling faster and more efficient decomposition.

Three mechanisms of UV-mediated organic decomposition are outlined in Figure 9, in which contribution to titanium-mediated photocatalysis was remarkably smaller than that of direct decomposition induced by UV light (photochemical and photophysical decomposition in Figure 9) for all light sources tested. Titanium is a semiconductor, so its photocatalytic activity is induced by UVA at wavelengths of ~375 nm, whose energy corresponds to the band gap of 3.2 eV on TiO_2_ surfaces to release e^−^ to the conduction band. The longer wavelength of UVA affords it higher permeability than UVB and UVC. Therefore, the photocatalytic contribution of UVC might be due to the relatively low intensity of UVC used in this study absorbed by the atmosphere and water, while the majority of UVA reached the titanium surfaces. As a result, UVA-triggered photocatalysis was pronounced. By contrast, the other UV sources of HUVC, PUV, and VUV, emitting high-intensity, short-wavelength UV rays, allowed the partial permeation of shorter UV rays through the atmosphere and water and less UVA-driven photocatalysis to be obtained. More importantly, our results suggest that photocatalytic decomposition is generally slow and has low efficiency and that direct decomposition induced by UV light is the most important parameter to consider when meeting requirements for clinical use (Figure 9). Previous studies have shown that 48 h of treatment is required to functionalize titanium using low-power bactericidal UV light [31,48,49].

We postulate that our finding that different materials and surfaces contributed little to the overall decomposition is due to the small contribution of photocatalysis to organic molecule decomposition. We conducted the experiment under the selected conditions using UVC and VUV light sources because information on light intensity was only available for these two sources. We selected the conditions in order to examine the effect of treatment time and type of container. There were no significant effects of different materials or surfaces under all conditions. In theory, the enlarged surface area of acid-etched microroughened titanium may increase photocatalysis. However, this must also be considered in the context of the different crystal properties of titanium surfaces processed in different ways. Heat-manufactured titanium dioxide particles form two crystal types, anatase and rutile; in general, anatase is stable at relatively low temperatures and more reactive to photocatalytic stimuli due to the favorable structure of the conduction band, while rutile is formed at high temperatures and is less photocatalytic [107,108,109,110,111,112,113]. Regular bulk titanium such as the machined surface used in this study is unlikely to have a crystal structure, but acid-etched titanium may have an altered crystal structure due to high-temperature acid etching. Future studies should further explore these material structures, including the effect of grade 5 titanium alloy; nevertheless, the photocatalytic contribution appears to be a relatively small component of the rapid organic decomposition required for clinical applications.

High-energy 172 nm VUV should be able to break strong carbon bonds. As shown in the photophysical decomposition illustrated in Figure 9, for instance, oxygen double bonds (O=O) and carbon double bonds (C=C) can be dissociated by 243 nm and 204 nm UV. However, UVC, with a peak wavelength of 254 nm, cannot break them. Thus, shorter wavelengths should decompose more molecules. Methylene blue is a synthetic, organic chloride salt (C_16_H_18_ClN_3_S) containing many carbon double bonds in their benzene rings, making degradation very difficult [97,114]. In light of this, the remarkable capability of VUV to decompose methylene blue and its linear load-testing capacity are of particular note. In photochemical decomposition (Figure 9), reactive oxygen species (ROS) generated by UV play an essential role. Although a low-pressure mercury lamp can generate ROS-excited atomic oxygen, O(^1^D), through the production of ozone O_3_, VUV excimer lamps can also generate O(^1^D) directly from O_2_ with a higher efficiency in addition to ozone-mediated generation, providing another explanation for the rapid and efficient VUV-mediated decomposition seen here.

One of the objectives of this study was to find a peak of decarbonization for VUV treatment. In light of this, as mentioned earlier, the one-minute treatment optimized in the dose-dependency experiment was more satisfactory than we anticipated, while the UVC light source took 20–30 min to accomplish the same. We conducted a dose-dependency test for HUVC and PUV sources but had to discontinue because the methylene blue solution started to evaporate after 1.5–3 min. Future studies using a dry technique instead of the wet technique used in this study need to be planned. More importantly, VUV is known to generate less heat than UVC, which has been demonstrated in this study and can be another advantage of VUV in clinical applications over other UV sources.

In this study, hydrophilicity/hydrophobicity and organic decomposition outcomes were not closely related. The efficacy of methylene blue removal varied considerably between the different UV light sources tested, while hydrophilic conversion was not significantly different. In fact, all light sources except for UVC converted titanium surfaces from hydrophobic to very hydrophilic or superhydrophilic (defined as a contact angle of 0°). This confirmed the importance of surface carbon rather than hydrophilicity as a marker for osseointegration, as reported previously [20,23,47,49]. Indeed, residual methylene blue varied between 75% and 9% on hydrophilic surfaces. We propose that surface hydrophilicity is an early marker of titanium photofunctionalization, i.e., hydrophilicity is a necessary but not sufficient factor for improved titanium bioactivity. More significantly, VUV treatment resulted in maximal hydrophilicity and organic decomposition within a minute.

## 4. Materials and Methods

### 4.1. Titanium Specimens and Surface Characterization

**Figure 10:**
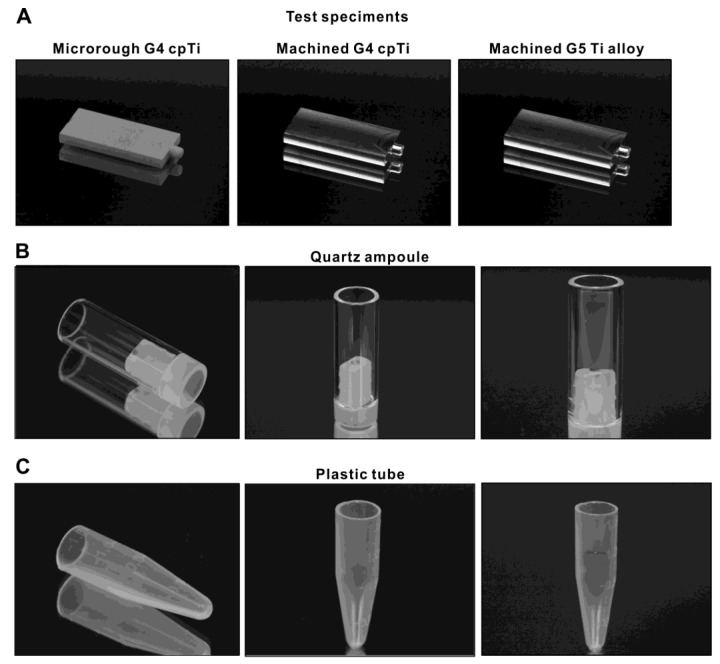
The titanium specimens and containers used in this study. (**A**) Test specimens made of different materials and surface topographies. (**B**) An ampoule made of synthetic quartz. A titanium specimen is placed in the quartz ampoule (**right** panel). (**C**) A laboratory-grade clear plastic tube. A titanium specimen is placed in the plastic tube (**right** panel).

Titanium test specimens in rectangular plate form (14 mm × 6 mm, 2 mm in thickness) were machined from commercially pure grade 4 titanium and grade 5 Ti-6Al-4V alloy (Figure 10A). To modify the surface from machine-smooth to microroughened, grade 4 commercially pure titanium plates were sandblasted and acid-etched. All test specimens were prepared and provided by DIO (Busan, Korea). Surface morphology was examined using scanning electron microscopy (SEM; Nova 230 Nano SEM; FEI, Hillsboro, Oregon). The hydrophilicity/hydrophobicity of titanium surfaces with and without UV treatment was evaluated by measuring the contact angle of 3 mL of ddH_2_O.

### 4.2. Methylene Blue as a Model Organic Molecule and Containers

Methylene blue was used as a model organic molecule to decompose using UV light. A 0.002% stock solution was prepared. Quartz ampoules in hollow cylinder form (10 mm in diameter, 25 mm in height, 1 mm thick) made from synthetic quartz glass (Figure 10B) and laboratory-grade 1.5 mL plastic tubes (Fisher Scientific, Pittsburgh, PA, USA) (Figure 10C) were prepared as containers for methylene blue solution during UV treatment. Quartz ampoules or plastic tubes were filled with 750 mL of methylene blue stock solution for UV treatment. UV treatment was administered with and without a titanium specimen in each container.

### 4.3. UV-Mediated Decomposition of Methylene Blue

**Figure 11:**
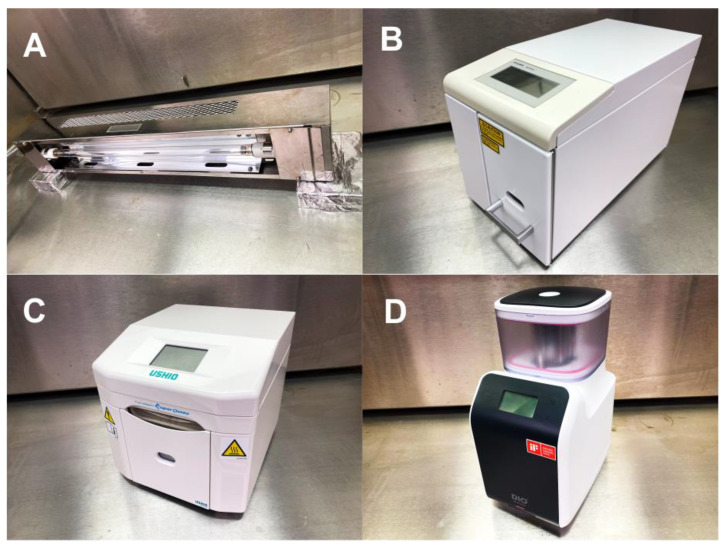
Photographs of UV devices used in this study. (**A**) UVC device (UVC). (**B**) High-energy UVC (HUVC). (**C**) UV device with a proprietary protocol (PUV). (**D**) 172 nm vacuum UV (VUV).

Methylene blue in quartz and plastic containers was treated with four different UV light sources (Figure 11): (i) UVC from a commercially available low-pressure mercury lamp (1.2 mW/cm^2^; Iwasaki Electric, Tokyo, Japan); (ii) high-energy UVC (HUVC) from a commercially available UV device for dental implants (TheraBeam Affiny, Ushio, Tokyo, Japan); (iii) a commercially available UV device for dental implants with a proprietary protocol (PUV) (SuperOsseo, Ushio, Tokyo, Japan); and (iv) 172 nm vacuum UV (VUV; ~60 mW/cm^2^) (DIO, Busan, Korea). UVC and VUV were irradiated at 6 mm and HUVC and PUV following the manufacturers’ instructions. The treatment time was 1 min for most experiments and was varied for dose-dependency experiments. The methylene blue concentrations in the original solution and after UV treatment were measured using a microplate reader at 650 nm (Synergy H1; BioTek Instruments, Winooski, VT, USA), and remnant methylene blue was calculated as a percentage relative to the original solution.

### 4.4. Statistical Analyses

Three test specimens were used for all methylene blue decomposition experiments (*n* = 3). One- or two-way ANOVA was performed to examine the effect of different UV light sources, the presence or absence of a titanium specimen, and treatment time. The Bonferroni test was used as a post hoc multiple comparison test where appropriate. *p*-values < 0.05 were considered to be statistically significant. Regression analysis was applied to determine the associations between methylene blue decomposition parameters and UV treatment factors.

## 5. Conclusions

Here, we determined the capability of a novel xenon excimer-generated VUV light source to decompose organic molecules around titanium, with a view to optimizing the photofunctionalization or activation of titanium implants for clinical use. VUV decomposed methylene blue molecules in a quartz ampoule significantly faster and with greater efficacy than other UV light sources, including low-pressure mercury-generated UVC and commercially available UV devices for dental implants. We achieved >90% VUV-mediated organic decomposition in one minute. Load testing revealed that VUV light decomposed more methylene blue as it was more concentrated, whereas there was a limit to the decomposing capacity of other UV light sources. All UV light sources tested here generated hydrophilic titanium surfaces after one minute of treatment. Thus, the ability and capacity of different UV light sources to induce organic decomposition varied considerably, with the fastest and most effective decomposition being achieved with VUV, paving the way for VUV-mediated photofunctionalization protocols and devices in clinical practice.

## Data Availability

The data presented in this study are available upon request from the corresponding author.

## References

[1] Tsukimura N., Ueno T., Iwasa F., Minamikawa H., Sugita Y., Ishizaki K., Ikeda T., Nakagawa K., Yamada M., Ogawa T. (2011). Bone integration capability of alkali- and heat-treated nanobimorphic Ti-15Mo-5Zr-3Al. Acta Biomater..

[2] Hasegawa M., Saruta J., Hirota M., Taniyama T., Sugita Y., Kubo K., Ishijima M., Ikeda T., Maeda H., Ogawa T. (2020). A Newly Created Meso-, Micro-, and Nano-Scale Rough Titanium Surface Promotes Bone-Implant Integration. Int. J. Mol. Sci..

[3] Ueno T., Tsukimura N., Yamada M., Ogawa T. (2011). Enhanced bone-integration capability of alkali- and heat-treated nanopolymorphic titanium in micro-to-nanoscale hierarchy. Biomaterials.

[4] Saruta J., Sato N., Ishijima M., Okubo T., Hirota M., Ogawa T. (2019). Disproportionate Effect of Sub-Micron Topography on Osteoconductive Capability of Titanium. Int. J. Mol. Sci..

[5] Saruta J., Ozawa R., Okubo T., Taleghani S.R., Ishijima M., Kitajima H., Hirota M., Ogawa T. (2021). Biomimetic Zirconia with Cactus-Inspired Meso-Scale Spikes and Nano-Trabeculae for Enhanced Bone Integration. Int. J. Mol. Sci..

[6] Takeuchi K., Saruwatari L., Nakamura H.K., Yang J.M., Ogawa T. (2005). Enhanced intrinsic biomechanical properties of osteoblastic mineralized tissue on roughened titanium surface. J. Biomed. Mater. Res. A.

[7] Tsukimura N., Kojima N., Kubo K., Att W., Takeuchi K., Kameyama Y., Maeda H., Ogawa T. (2008). The effect of superficial chemistry of titanium on osteoblastic function. J. Biomed. Mater. Res. A.

[8] Nakamura H., Saruwatari L., Aita H., Takeuchi K., Ogawa T. (2005). Molecular and biomechanical characterization of mineralized tissue by dental pulp cells on titanium. J. Dent. Res..

[9] Nakamura H., Shim J., Butz F., Aita H., Gupta V., Ogawa T. (2006). Glycosaminoglycan degradation reduces mineralized tissue-titanium interfacial strength. J. Biomed. Mater. Res. A.

[10] Nakamura H.K., Butz F., Saruwatari L., Ogawa T. (2007). A role for proteoglycans in mineralized tissue-titanium adhesion. J. Dent. Res..

[11] Saruwatari L., Aita H., Butz F., Nakamura H.K., Ouyang J., Yang Y., Chiou W.A., Ogawa T. (2005). Osteoblasts generate harder, stiffer, and more delamination-resistant mineralized tissue on titanium than on polystyrene, associated with distinct tissue micro- and ultrastructure. J. Bone Min. Res..

[12] Kubo K., Tsukimura N., Iwasa F., Ueno T., Saruwatari L., Aita H., Chiou W.A., Ogawa T. (2009). Cellular behavior on TiO_2_ nanonodular structures in a micro-to-nanoscale hierarchy model. Biomaterials.

[13] Att W., Kubo K., Yamada M., Maeda H., Ogawa T. (2009). Biomechanical properties of jaw periosteum-derived mineralized culture on different titanium topography. Int. J. Oral Maxillofac. Implant..

[14] Att W., Tsukimura N., Suzuki T., Ogawa T. (2007). Effect of supramicron roughness characteristics produced by 1- and 2-step acid etching on the osseointegration capability of titanium. Int. J. Oral Maxillofac. Implant..

[15] Ishizaki K., Sugita Y., Iwasa F., Minamikawa H., Ueno T., Yamada M., Suzuki T., Ogawa T. (2011). Nanometer-thin TiO(2) enhances skeletal muscle cell phenotype and behavior. Int. J. Nanomed..

[16] Kojima N., Ozawa S., Miyata Y., Hasegawa H., Tanaka Y., Ogawa T. (2008). High-throughput gene expression analysis in bone healing around titanium implants by DNA microarray. Clin. Oral Implant. Res..

[17] Rowlands D.S., Shultz S.P., Ogawa T., Aoi W., Korte M. (2014). The effects of uniquely-processed titanium on biological systems: Implications for human health and performance. J. Funct. Biomater..

[18] Sugita Y., Ishizaki K., Iwasa F., Ueno T., Minamikawa H., Yamada M., Suzuki T., Ogawa T. (2011). Effects of pico-to-nanometer-thin TiO_2_ coating on the biological properties of microroughened titanium. Biomaterials.

[19] Hori N., Iwasa F., Ueno T., Takeuchi K., Tsukimura N., Yamada M., Hattori M., Yamamoto A., Ogawa T. (2010). Selective cell affinity of biomimetic micro-nano-hybrid structured TiO_2_ overcomes the biological dilemma of osteoblasts. Dent. Mater. Off. Publ. Acad. Dent. Mater..

[20] Att W., Hori N., Takeuchi M., Ouyang J., Yang Y., Anpo M., Ogawa T. (2009). Time-dependent degradation of titanium osteoconductivity: An implication of biological aging of implant materials. Biomaterials.

[21] Att W., Ogawa T. (2012). Biological aging of implant surfaces and their restoration with ultraviolet light treatment: A novel understanding of osseointegration. Int. J. Oral Maxillofac. Implant..

[22] Hori N., Att W., Ueno T., Sato N., Yamada M., Saruwatari L., Suzuki T., Ogawa T. (2009). Age-dependent degradation of the protein adsorption capacity of titanium. J. Dent. Res..

[23] Hori N., Ueno T., Suzuki T., Yamada M., Att W., Okada S., Ohno A., Aita H., Kimoto K., Ogawa T. (2010). Ultraviolet light treatment for the restoration of age-related degradation of titanium bioactivity. Int. J. Oral Maxillofac. Implant..

[24] Suzuki T., Hori N., Att W., Kubo K., Iwasa F., Ueno T., Maeda H., Ogawa T. (2009). Ultraviolet treatment overcomes time-related degrading bioactivity of titanium. Tissue Eng. Part A.

[25] Suzuki T., Kubo K., Hori N., Yamada M., Kojima N., Sugita Y., Maeda H., Ogawa T. (2010). Nonvolatile buffer coating of titanium to prevent its biological aging and for drug delivery. Biomaterials.

[26] Ueno T., Takeuchi M., Hori N., Iwasa F., Minamikawa H., Igarashi Y., Anpo M., Ogawa T. (2012). Gamma ray treatment enhances bioactivity and osseointegration capability of titanium. J. Biomed. Mater. Research. Part B Appl. Biomater..

[27] Hirota M., Hori N., Sugita Y., Ikeda T., Park W., Saruta J., Ogawa T. (2022). A Novel Cell Delivery System Exploiting Synergy between Fresh Titanium and Fibronectin. Cells.

[28] Iwasa F., Tsukimura N., Sugita Y., Kanuru R.K., Kubo K., Hasnain H., Att W., Ogawa T. (2011). TiO_2_ micro-nano-hybrid surface to alleviate biological aging of UV-photofunctionalized titanium. Int. J. Nanomed..

[29] Nakhaei K., Ishijima M., Ikeda T., Ghassemi A., Saruta J., Ogawa T. (2020). Ultraviolet Light Treatment of Titanium Enhances Attachment, Adhesion, and Retention of Human Oral Epithelial Cells via Decarbonization. Materials.

[30] Hayashi R., Ueno T., Migita S., Tsutsumi Y., Doi H., Ogawa T., Hanawa T., Wakabayashi N. (2014). Hydrocarbon Deposition Attenuates Osteoblast Activity on Titanium. J. Dent. Res..

[31] Att W., Hori N., Iwasa F., Yamada M., Ueno T., Ogawa T. (2009). The effect of UV-photofunctionalization on the time-related bioactivity of titanium and chromium-cobalt alloys. Biomaterials.

[32] Ikeda T., Hagiwara Y., Hirota M., Tabuchi M., Yamada M., Sugita Y., Ogawa T. (2014). Effect of photofunctionalization on fluoride-treated nanofeatured titanium. J. Biomater. Appl..

[33] Ikeda T., Okubo T., Saruta J., Hirota M., Kitajima H., Yanagisawa N., Ogawa T. (2021). Osteoblast Attachment Compromised by High and Low Temperature of Titanium and Its Restoration by UV Photofunctionalization. Materials.

[34] Ikeda T., Ueno T., Saruta J., Hirota M., Park W., Ogawa T. (2021). Ultraviolet Treatment of Titanium to Enhance Adhesion and Retention of Oral Mucosa Connective Tissue and Fibroblasts. Int. J. Mol. Sci..

[35] Iwasaki C., Hirota M., Tanaka M., Kitajima H., Tabuchi M., Ishijima M., Park W., Sugita Y., Miyazawa K., Goto S. (2020). Tuning of Titanium Microfiber Scaffold with UV-Photofunctionalization for Enhanced Osteoblast Affinity and Function. Int. J. Mol. Sci..

[36] Minamikawa H., Att W., Ikeda T., Hirota M., Ogawa T. (2016). Long-Term Progressive Degradation of the Biological Capability of Titanium. Materials.

[37] Okubo T., Ikeda T., Saruta J., Tsukimura N., Hirota M., Ogawa T. (2020). Compromised Epithelial Cell Attachment after Polishing Titanium Surface and Its Restoration by UV Treatment. Materials.

[38] Saita M., Ikeda T., Yamada M., Kimoto K., Lee M.C., Ogawa T. (2016). UV photofunctionalization promotes nano-biomimetic apatite deposition on titanium. Int. J. Nanomed..

[39] Sugita Y., Saruta J., Taniyama T., Kitajima H., Hirota M., Ikeda T., Ogawa T. (2020). UV-Pre-Treated and Protein-Adsorbed Titanium Implants Exhibit Enhanced Osteoconductivity. Int. J. Mol. Sci..

[40] Tabuchi M., Ikeda T., Hirota M., Nakagawa K., Park W., Miyazawa K., Goto S., Ogawa T. (2015). Effect of UV Photofunctionalization on Biologic and Anchoring Capability of Orthodontic Miniscrews. Int. J. Oral Maxillofac. Implant..

[41] Tabuchi M., Ikeda T., Nakagawa K., Hirota M., Park W., Miyazawa K., Goto S., Ogawa T. (2015). Ultraviolet photofunctionalization increases removal torque values and horizontal stability of orthodontic miniscrews. Am J. Orthod. Dentofac. Orthop..

[42] Taniyama T., Saruta J., Mohammadzadeh Rezaei N., Nakhaei K., Ghassemi A., Hirota M., Okubo T., Ikeda T., Sugita Y., Hasegawa M. (2020). UV-Photofunctionalization of Titanium Promotes Mechanical Anchorage in A Rat Osteoporosis Model. Int. J. Mol. Sci..

[43] Ueno T., Ikeda T., Tsukimura N., Ishijima M., Minamikawa H., Sugita Y., Yamada M., Wakabayashi N., Ogawa T. (2016). Novel antioxidant capability of titanium induced by UV light treatment. Biomaterials.

[44] Hirota M., Ikeda T., Sugita Y., Ishijima M., Hirota S., Ogawa T. (2019). Impaired osteoblastic behavior and function on saliva-contaminated titanium and its restoration by UV treatment. Mater. Sci. Eng. C Mater. Biol. Appl..

[45] Hirota M., Ikeda T., Tabuchi M., Iwai T., Tohnai I., Ogawa T. (2014). Effect of ultraviolet-mediated photofunctionalization for bone formation around medical titanium mesh. J. Oral Maxillofac. Surg..

[46] Hirota M., Ikeda T., Tabuchi M., Nakagawa K., Park W., Ishijima M., Tsukimura N., Hagiwara Y., Ogawa T. (2016). Bone Generation Profiling Around Photofunctionalized Titanium Mesh. Int. J. Oral Maxillofac. Implant..

[47] Hirota M., Sugita Y., Ishijima M., Ikeda T., Saruta J., Maeda H., Ogawa T. (2021). UV photocatalytic activity of titanium dioxide (TiO_2_) surface contaminated with bacterial biofilm: Implications for photo- restoration of osteoconductivity. Mater. Today Adv..

[48] Aita H., Att W., Ueno T., Yamada M., Hori N., Iwasa F., Tsukimura N., Ogawa T. (2009). Ultraviolet light-mediated photofunctionalization of titanium to promote human mesenchymal stem cell migration, attachment, proliferation and differentiation. Acta Biomater..

[49] Aita H., Hori N., Takeuchi M., Suzuki T., Yamada M., Anpo M., Ogawa T. (2009). The effect of ultraviolet functionalization of titanium on integration with bone. Biomaterials.

[50] Ogawa T. (2014). Ultraviolet photofunctionalization of titanium implants. Int. J. Oral Maxillofac. Implant..

[51] Funato A., Ogawa T. (2013). Photofunctionalized dental implants: A case series in compromised bone. Int. J. Oral Maxillofac. Implant..

[52] Funato A., Yamada M., Ogawa T. (2013). Success rate, healing time, and implant stability of photofunctionalized dental implants. Int. J. Oral Maxillofac. Implant..

[53] Hirota M., Ozawa T., Iwai T., Ogawa T., Tohnai I. (2018). Effect of Photofunctionalization on Early Implant Failure. Int. J. Oral Maxillofac. Implant..

[54] Hirota M., Tanaka M., Ishijima M., Iwasaki C., Park W., Ogawa T. (2016). Effect of Photofunctionalization on Ti6Al4V Screw Stability Placed in Segmental Bone Defects in Rat Femurs. J. Oral Maxillofac. Surg..

[55] Hori N., Iwasa F., Tsukimura N., Sugita Y., Ueno T., Kojima N., Ogawa T. (2011). Effects of UV photofunctionalization on the nanotopography enhanced initial bioactivity of titanium. Acta Biomater..

[56] Hori N., Ueno T., Minamikawa H., Iwasa F., Yoshino F., Kimoto K., Lee M.C., Ogawa T. (2010). Electrostatic control of protein adsorption on UV-photofunctionalized titanium. Acta Biomater..

[57] Iwasa F., Baba K., Ogawa T. (2016). Enhanced intracellular signaling pathway in osteoblasts on ultraviolet lighttreated hydrophilic titanium. Biomed. Res..

[58] Iwasa F., Hori N., Ueno T., Minamikawa H., Yamada M., Ogawa T. (2010). Enhancement of osteoblast adhesion to UV-photofunctionalized titanium via an electrostatic mechanism. Biomaterials.

[59] Jokstad A., Sanz M., Ogawa T., Bassi F., Levin L., Wennerberg A., Romanos G.E. (2016). A Systematic Review of the Role of Implant Design in the Rehabilitation of the Edentulous Maxilla. Int. J. Oral Maxillofac. Implant..

[60] Kitajima H., Ogawa T. (2016). The Use of Photofunctionalized Implants for Low or Extremely Low Primary Stability Cases. Int. J. Oral Maxillofac. Implant..

[61] Lee J.H., Ogawa T. (2012). The biological aging of titanium implants. Implant Dent..

[62] Minamikawa H., Ikeda T., Att W., Hagiwara Y., Hirota M., Tabuchi M., Aita H., Park W., Ogawa T. (2014). Photofunctionalization increases the bioactivity and osteoconductivity of the titanium alloy Ti6Al4V. J. Biomed. Mater. Res. A.

[63] Suzuki S., Kobayashi H., Ogawa T. (2013). Implant stability change and osseointegration speed of immediately loaded photofunctionalized implants. Implant Dent..

[64] Al Qahtani M.S., Wu Y., Spintzyk S., Krieg P., Killinger A., Schweizer E., Stephan I., Scheideler L., Geis-Gerstorfer J., Rupp F. (2015). UV-A and UV-C light induced hydrophilization of dental implants. Dent. Mater..

[65] Altmann B., Kohal R.J., Steinberg T., Tomakidi P., Bachle-Haas M., Wennerberg A., Att W. (2013). Distinct cell functions of osteoblasts on UV-functionalized titanium- and zirconia-based implant materials are modulated by surface topography. Tissue Eng. Part C Methods.

[66] Choi B., Lee Y.C., Oh K.C., Lee J.H. (2021). Effects of photofunctionalization on early osseointegration of titanium dental implants in the maxillary posterior region: A randomized double-blinded clinical trial. Int. J. Implant Dent..

[67] de Avila E.D., Lima B.P., Sekiya T., Torii Y., Ogawa T., Shi W., Lux R. (2015). Effect of UV-photofunctionalization on oral bacterial attachment and biofilm formation to titanium implant material. Biomaterials.

[68] Flanagan D. (2016). Photofunctionalization of Dental Implants. J. Oral Implant..

[69] Gao Y., Liu Y., Zhou L., Guo Z., Rong M., Liu X., Lai C., Ding X. (2013). The effects of different wavelength UV photofunctionalization on micro-arc oxidized titanium. PLoS ONE.

[70] Ghassemi A., Ishijima M., Hasegawa M., Mohammadzadeh Rezaei N., Nakhaei K., Sekiya T., Torii Y., Hirota M., Park W., Miley D.D. (2018). Biological and Physicochemical Characteristics of 2 Different Hydrophilic Surfaces Created by Saline-Storage and Ultraviolet Treatment. Implant Dent..

[71] Park K.H., Koak J.Y., Kim S.K., Heo S.J. (2011). Wettability and cellular response of UV light irradiated anodized titanium surface. J. Adv. Prosthodont..

[72] Pyo S.W., Park Y.B., Moon H.S., Lee J.H., Ogawa T. (2013). Photofunctionalization enhances bone-implant contact, dynamics of interfacial osteogenesis, marginal bone seal, and removal torque value of implants: A dog jawbone study. Implant Dent..

[73] Ueno T., Yamada M., Suzuki T., Minamikawa H., Sato N., Hori N., Takeuchi K., Hattori M., Ogawa T. (2010). Enhancement of bone-titanium integration profile with UV-photofunctionalized titanium in a gap healing model. Biomaterials.

[74] Att W., Takeuchi M., Suzuki T., Kubo K., Anpo M., Ogawa T. (2009). Enhanced osteoblast function on ultraviolet light-treated zirconia. Biomaterials.

[75] Sugita Y., Honda Y., Kato I., Kubo K., Maeda H., Ogawa T. (2014). Role of photofunctionalization in mitigating impaired osseointegration associated with type 2 diabetes in rats. Int. J. Oral Maxillofac. Implant..

[76] Ogawa T., Kamat P., Anpo M. (2010). Photofunctionalization of TiO_2_ for optimal integration of titanium with bone. Benign Photocatalysts. Applications of Titanium Oxide-Based Materials.

[77] Miyauchi T., Yamada M., Yamamoto A., Iwasa F., Suzawa T., Kamijo R., Baba K., Ogawa T. (2010). The enhanced characteristics of osteoblast adhesion to photofunctionalized nanoscale TiO_2_ layers on biomaterials surfaces. Biomaterials.

[78] Yamada M., Miyauchi T., Yamamoto A., Iwasa F., Takeuchi M., Anpo M., Sakurai K., Baba K., Ogawa T. (2010). Enhancement of adhesion strength and cellular stiffness of osteoblasts on mirror-polished titanium surface by UV-photofunctionalization. Acta Biomater..

[79] Hirota M., Ikeda T., Tabuchi M., Ozawa T., Tohnai I., Ogawa T. (2017). Effects of Ultraviolet Photofunctionalization on Bone Augmentation and Integration Capabilities of Titanium Mesh and Implants. Int. J. Oral Maxillofac. Implant..

[80] Hirota M., Ozawa T., Iwai T., Mitsudo K., Ogawa T. (2020). UV-Mediated Photofunctionalization of Dental Implant: A Seven-Year Results of a Prospective Study. J. Clin. Med..

[81] Aita H., Oh W., Kubo K., Tsukimura N., Maeda H., Ogawa T. (2008). Light-induced bone cement-philic titanium surface. J. Mater. Sci..

[82] Kitajima H., Hirota M., Iwai T., Hamajima K., Ozawa R., Hayashi Y., Yajima Y., Iida M., Koizumi T., Kioi M. (2020). Computational Fluid Simulation of Fibrinogen around Dental Implant Surfaces. Int. J. Mol. Sci..

[83] Funato A., Tonotsuka R., Murabe H., Hirota M., Ogawa T. (2014). A novel strategy for bone integration and regeneration-Photofunctionalization of dental implants and Ti mesh. J. Cosmet. Dent..

[84] Ishikawa T., Salama M., Funato A., Kitajima H., Moroi H., Salama H., Garber D. (2010). Three-dimensional bone and soft tissue requirements for optimizing esthetic results in compromised cases with multiple implants. Int. J. Periodontics Restor. Dent..

[85] Hirota M., Ozawa T., Iwai T., Ogawa T., Tohnai I. (2016). Implant Stability Development of Photofunctionalized Implants Placed in Regular and Complex Cases: A Case-Control Study. Int. J. Oral Maxillofac. Implant..

[86] Ishikawa T., Vela X., Kida K., Moroi H., Kitajima H., Ogawa T. (2014). Restoration of optimum esthetics in complex clinical situations using an interdisciplinary strategy in combination with advanced techniques and technologies in regenerative medicine. J. Cosmet. Dent..

[87] Takeuchi M., Martra G., Coluccia S., Anpo M. (2005). Investigations of the structure of H_2_O clusters adsorbed on TiO_2_ surfaces by near-infrared absorption spectroscopy. J. Phys. Chem. B.

[88] Takeuchi M., Sakamoto K., Martra G., Coluccia S., Anpo M. (2005). Mechanism of photoinduced superhydrophilicity on the TiO_2_ photocatalyst surface. J. Phys. Chem. B.

[89] Chen H., Nanayakkara C.E., Grassian V.H. (2012). Titanium dioxide photocatalysis in atmospheric chemistry. Chem. Rev..

[90] Gopinath K.P., Madhav N.V., Krishnan A., Malolan R., Rangarajan G. (2020). Present applications of titanium dioxide for the photocatalytic removal of pollutants from water: A review. J. Environ. Manag..

[91] Wang R., Hashimoto K., Fujishima A. (1997). Light-induced amphiphilic surfaces. Nature.

[92] Gyorgyey A., Janovak L., Adam A., Kopniczky J., Toth K.L., Deak A., Panayotov I., Cuisinier F., Dekany I., Turzo K. (2016). Investigation of the in vitro photocatalytic antibacterial activity of nanocrystalline TiO_2_ and coupled TiO_2_/Ag containing copolymer on the surface of medical grade titanium. J. Biomater. Appl..

[93] Ballman A.A., Dodd D.M., Kuebler N.A., Laudise R.A., Wood D.L., Rudd D.W. (1968). Synthetic quartz with high ultraviolet transmission. Appl. Opt..

[94] Donat C.P. (1945). Ultraviolet radiation from quartz lamps. Rev. Radiol. Fisioter.

[95] Lei D., Xie X., Xiang Y., Huang X., Xiao F., Cao J., Li G., Leung D.Y.C., Huang H. (2022). An efficient process for aromatic VOCs degradation: Combination of VUV photolysis and photocatalytic oxidation in a wet scrubber. Chemosphere.

[96] Zaplotnik R., Vesel A. (2020). Effect of VUV Radiation on Surface Modification of Polystyrene Exposed to Atmospheric Pressure Plasma Jet. Polymers.

[97] Wen D., Li W., Lv J., Qiang Z., Li M. (2020). Methylene blue degradation by the VUV/UV/persulfate process: Effect of pH on the roles of photolysis and oxidation. J. Hazard. Mater..

[98] Fu P., Ma Y., Lei B., Li G., Lin X. (2021). Decomposition of refractory aniline aerofloat collector in aqueous solution by an ozone/vacuum-UV (O3/VUV) process. Environ. Technol..

[99] Long L., Bu Y., Chen B., Sadiq R. (2019). Removal of urea from swimming pool water by UV/VUV: The roles of additives, mechanisms, influencing factors, and reaction products. Water Res..

[100] Park W., Ishijima M., Hirota M., Soltanzadeh P., Ogawa T. (2016). Engineering bone-implant integration with photofunctionalized titanium microfibers. J. Biomater. Appl..

[101] Tateshima S., Kaneko N., Yamada M., Duckwiler G., Vinuela F., Ogawa T. (2018). Increased affinity of endothelial cells to NiTi using ultraviolet irradiation: An in vitro study. J. Biomed. Mater. Res. A.

[102] Bono N., Ponti F., Punta C., Candiani G. (2021). Effect of UV Irradiation and TiO_2_-Photocatalysis on Airborne Bacteria and Viruses: An Overview. Materials.

[103] Bertagna Silva D., Buttiglieri G., Babic S. (2021). State-of-the-art and current challenges for TiO_2_/UV-LED photocatalytic degradation of emerging organic micropollutants. Environ. Sci. Pollut. Res. Int..

[104] Iervolino G., Zammit I., Vaiano V., Rizzo L. (2019). Limitations and Prospects for Wastewater Treatment by UV and Visible-Light-Active Heterogeneous Photocatalysis: A Critical Review. Top. Curr. Chem..

[105] Matafonova G., Batoev V. (2018). Recent advances in application of UV light-emitting diodes for degrading organic pollutants in water through advanced oxidation processes: A review. Water Res..

[106] Egerton T.A. (2014). UV-absorption--the primary process in photocatalysis and some practical consequences. Molecules.

[107] Jing J., Feng J., Li W., Yu W.W. (2013). Low-temperature synthesis of water-dispersible anatase titanium dioxide nanoparticles for photocatalysis. J. Colloid Interface Sci..

[108] Li W., Bai Y., Liu C., Yang Z., Feng X., Lu X., van der Laak N.K., Chan K.Y. (2009). Highly thermal stable and highly crystalline anatase TiO_2_ for photocatalysis. Environ. Sci. Technol..

[109] Lee K., Kim D., Roy P., Paramasivam I., Birajdar B.I., Spiecker E., Schmuki P. (2010). Anodic formation of thick anatase TiO_2_ mesosponge layers for high-efficiency photocatalysis. J. Am Chem.. Soc..

[110] Liu M., Piao L., Zhao L., Ju S., Yan Z., He T., Zhou C., Wang W. (2010). Anatase TiO(2) single crystals with exposed {001} and {110} facets: Facile synthesis and enhanced photocatalysis. Chem. Commun..

[111] Setvin M., Shi X., Hulva J., Simschitz T., Parkinson G.S., Schmid M., Di Valentin C., Selloni A., Diebold U. (2017). Methanol on Anatase TiO_2_ (101): Mechanistic Insights into Photocatalysis. ACS Catal.

[112] Sun B., Smirniotis P.G., Boolchand P. (2005). Visible light photocatalysis with platinized rutile TiO_2_ for aqueous organic oxidation. Langmuir.

[113] Walenta C.A., Kollmannsberger S.L., Kiermaier J., Winbauer A., Tschurl M., Heiz U. (2015). Ethanol photocatalysis on rutile TiO_2_(110): The role of defects and water. Phys. Chem. Chem. Phys..

[114] Liu C., Yang B., Chen J., Jia F., Song S. (2022). Synergetic degradation of Methylene Blue through photocatalysis and Fenton reaction on two-dimensional molybdenite-Fe. J. Environ. Sci..

